# On the Treatment of Scabies

**Published:** 1846-04

**Authors:** 


12. *On the treatment of Scabies.*—Every practitioner has had occasion to lament that the best remedy for this disease is one of so disgusting a nature as sulphur ointment. The profession, therefore, will be glad to learn from the following communication from Mr. Stiff, that the sulphur may be dispensed with, and that the essential portion of the compound is the adipose matter. Mr. Stiff states, that during the last six months he has frequently tested the action of sulphur in scabies, and considers that it may be cured without it. The truth of this assertion is rendered more evident, he thinks, by looking into the nature of the disease, and by examining the anatomy of the animal. The acarus scabiei belongs to a class of insects which breathe by the tracheæ, and its respiration is therefore suspended by smearing its body with any oleaginous substance. It is in this manner, then, the sulphur ointment acts, according to the author, and not in virtue of any specific action of the sulphur. In proof of this, he affirms that he has cured more than forty cases by inunction with simple lard, unaided by any other treatment. The average duration of treatment was only a week.—*Med. Times*, July 26, 1845, in *St. Louis Med. and Surg. Journal.*

